# High-matrix-stiffness induces promotion of hepatocellular carcinoma proliferation and suppression of apoptosis via miR-3682-3p-PHLDA1-FAS pathway

**DOI:** 10.7150/jca.45998

**Published:** 2020-08-25

**Authors:** Bowen Yao, Yongshen Niu, Yazhao Li, Tianxiang Chen, Xinyu Wei, Qingguang Liu

**Affiliations:** 1 Department of Hepatobiliary Surgery, the First Affiliated Hospital of Xi'an Jiaotong University, No. 277 Yanta West Road, Xi'an 710061, China; 2 Center for Translational Medicine, the First Affiliated Hospital of Xi'an Jiaotong University, No. 277 Yanta West Road, Xi'an 710061, China; 3 Medicine college, Xi'an Jiaotong University, No. 76 Yanta West Road, Xi'an 710061, China

**Keywords:** MicroRNA-3682-3p, extracellular matrix stiffness, PHLDA1, apoptosis, hepatocellular carcinoma

## Abstract

Hepatocellular carcinoma (HCC) with malignant behaviors related to death causes distant metastasis and is the fourth primary cancer in the whole world, which has taken millions lives in Asian countries such as China. The novel miR-3682-3p involving high-expression-related poor prognosis in HCC tissues and cell lines indicate oncogenesis functions in vitro and in vivo. According to TCGA database, our group find several none-coding RNAs showing abnormal expression including miR-3682-3p, thus we originally confirmed the inhibition of proliferation and acceleration of apoptosis are enhanced in miR-3682-3p knock-down cell lines. Then, in nude mice transplantation assays, we found the suppressor behaviors, smaller nodules and lower speed of tumor expansion in model of injection of cell cultured and transfected shRNA-miR-3682-3p. A combination of databases (Starbase, Targetscan and MiRgator) illustrates miR-3682-3p targets PHLDA1, which shows negative correlation demonstrated by dual-luciferase reporter system. To make functional verification of PHLDA1, we upregulate the gene and rescue tests are established to confirm that miR-3682-3p suppresses PHLDA1 to promotion of cell growth. Rescue experiments finish making confirmation of relation of miR-3682-3p and PHLDA1 subsequently. Cirrhotic tissues illustrate strong correlation to higher miR-3682-3p and clinical features make the hint that high-extracellular-matrix-stiffness environment promotes such miRNA. Functional tests on different stiffness provide the proof of underlying mechanism. In conclusion, the overexpression of miR-3682-3p mediates PHLDA1 inhibition could impede apoptosis and elevate proliferation of HCC through high-extracellular-matrix-stiffness environment potentially.

## Introduction

Hepatocellular carcinoma ranks as fourth frequently diagnosed cancer in global surveys and the thirds deadly cause of China in 2018 national health survey 1. With high early local and systemic metastasis features, HCC became well-known in cancer research area and was difficult for more and more researchers to underestimate the significance and researching values of such cancer 2, 3. Regardless of the many efforts made toward the treatment of malignancies, HCC remains incurable owing to high metastatic potential, with most deaths from HCC being attributable to its aggressiveness and growing resistance to the existing targeted medicines 4, 5. Extracellular matrix stiffness (ECM) environment confirmed in many types of cancers is closely related to metastasis 6, apoptosis 7 and invasion 8 respectively, therefore, identifying pathways and down-streams of high ECM stiffness conditions might lead to new therapeutic candidates for HCC. Angiogenesis, reprogramming of cytoskeleton, extracellular matrix remodeling and promotion of inflationary cell chemotaxis turn out to be consequences of two or three dimensional high ECM stiffness 8, 9. However, the characteristics of HCC especially under the high ECM stiffness condition, have not been fully understood.

A number of microRNAs (miRNAs), including miR-21 10, 11 and miR-122 12, 13, have been widely studied in regard to invasive HCC. The miR-876^14^ and miR-1468^15^ along with others have been reported in various articles. Mentioning about high ECM stiffness, miR-26 and miR-29 must be the famous stars of messenger for mediate the LOX family members and activity of collagens 16, 17. In addition, miR-185 and miR-210 had been tested to regulate HCC behavior in high ECM stiffness 18, 19. Therefore, there is great potential for identifying an underlying miRNA that can be used to develop treatments for HCC and assist in early of HCC detection and the control of metastasis 20, 21.

miRNAs play an independent role in the pathogenesis and progression of almost all types of cancers, such as non-small cell lung cancer , breast cancer , gastric carcinoma, bladder tumors and may other known tumor types. They are responsible for modulation of their cellular differentiation, as well as in regulating the transcription of their downstream targets 22-29.

The novel miRNA, miR-3682-3p, has never been reported in any article about neoplasms, including HCC. With the support of advanced analysis of a considerable amount of HCC samples, we identified the new miR-3682-3p and investigated its function in HCC and in the high ECM stiffness in HCC.

Pleckstrin Homology Like Domain Family A Member 1 (PHLDA1) was initially discovered in 1999 and was known as TDAG5^30^, which structural prediction share a motif resembling a pleckstrin-homology (PH) domain with Tih1 and Tssc3, and was a gene involved in FAS/CD95 expression in human species 31. Before nearly 10 years, several studies had been established to make sure functional strategy of PHLDA1 in breast cancer and neuroblastoma, which was effector of aurora A kinase and led to cell death 32, 33. Apoptosis is a crucial phenomenon when cancer cell surfer from radio or chemical stimuli 34-37. The suppression of apoptosis behavior has been explored in oral cancer 38. However, in many species like rat, rabbit and human, PHLDA1 was a needy role for Fas/FasL expression, and inhibited Akt pathway in various tumor behaviors such as apoptosis, autophagy, epithelial to mesenchymal transition (EMT) and endoplasmic reticulum (ER) stress 30-33, 35, 39-41. In HCC tissues, about PHLDA1, there was no test on tumor features, except one predication gene result indicated PHLDA1 was low expressed in samples 42. In conclusion, there was no final verdict of the values whether PHLDA1 played a positive or negative status in HCC with the high-expression in tumor specimens.

Therefore, in this study, we evaluated the potential mechanism of cell growth producer and apoptosis inhibitor, miR-3682-3p, in HCC and found that it targeted PHLDA1 in high ECM stiffness condition.

## Materials and Methods

### Study objects

HCC tissues and adjacent tissues were obtained from 92 patients in the First Affiliated Hospital of Xi'an Jiaotong University from January 2012 to December 2016 for the current study. This study was approved by the Ethics Committee of the First Affiliated Hospital of Xi'an Jiaotong University and signed informed consents were obtained from all participants prior to the study. All patients with 48.4 ± 6.5 years old mean age, including 33 patients under the age of 50 years and 59 patients were older than another age group. A total of 76 male patients and 16 female patients were included in this study. According to Edmondson and tumor-node-metastasis stages, 39 patients in early stage and 53 patients in latter stage of Edmondson, and 48 in the early and 44 in the latter of TNM stages, respectively. Besides, tumor number, tumor size in centimeters, venous invasion, cirrhosis and alpha-fetoprotein (AFP) were collected and analyzed by statistical methods. Some indispensable inclusion criteria when choosing suitable patients for our further surveys were as follows: (1) the pathological diagnosis of HCC had been made; (2) no previous treatments involving radiotherapy, chemotherapy and immunotherapy had been admin surgery; (3) samples for researches only came from patients who had undergone liver resection for HCC; (4) complete clinical and pathological information as well as prognostic records were available.

### Extracellular matrix stiffness culture

Variable ratios of acrylamide/bisacrylamide were mixed with tetramethylethylenediamine, APS and SBS for polymerization according to the formula shown in **Figure S1A** to prepare polyacrylamide hydrogels with stiffness ranging from 0.4 kilopascal (kPa) to 25.6 kPa. Each gel mixture was then immediately pipetted on the center of a coverslip that was previously washed with 0.4% MPS solution for salinization. Another coverslip, previously treated with SurfafSil to form hydrophobic surface, was placed on top of the gel solution for 2 minutes. Then removed the top coverslip and treated the gel with dopamine hydrochloride solution (1 mg/mL in 50 mM HEPES, pH 8.5) for 20 minutes followed by incubation with collagen I solution (0.05 mg/mL in PBS, pH 7.4) for 30 minutes. 30 minutes later, rinsing each gel with PBS and sterilization by microwave heating, the polyacrylamide gels were then placed in a 6-well cell culture plate for cell seeding and the following experiments. Three independent replicates were finished in each group.

### Cell Cycle

MHCC-97H cells and SMMC-7721 cells were both left in different groups, like anti-NC and anti-3682-3p in **Figure 2**, which were cultured on common culture plates, or three groups as **Figure 6** (grouped in anti-NC+si-NC, anti-3682-3p+si-NC and anti-3682-3p+si-PHLDA1-2), or were cultured on 0.4kpa or 25.6kpa extracellular matrix stiffness shown in **Figure 7**. Cells were ready for digestion by Trypsin without EDTA and only cells were collected as sediments after three times table-top low speed centrifugation (later twice washes in 2% 4℃ FBS-PBS). Cells treated before were all stored in 70% ethanol at -20℃ at least 4 hours. All the cells washed by 2% 4℃ FBS-PBS were then stained with 0.5 mL PI/RNase staining buffer for 28 minutes at room temperature then analyzed by flow cytometry (BD, #FACS Calibur, USA). Three independent replicates were finished in each group.

### Cell culture

Cell lines MHCC-97H, Hep3B and SMMC-7721 (Shanghai Institute of Biochemistry and Cell Biology Chinese Academy of Sciences, Shanghai, China) were added with the Dulbecco's modified Eagle medium (DMEM)(#HyClone, GE, Buckinghamshire, England) conjugated with 10% fetal bovine serum (FBS) (#Gibco, ThermoFisher, Shanghai, China) as complete medium and incubated in 5% CO2 and 37℃ surrounding. Other cell lines of HCC like Huh7 and MHCC-97L were tested in form of cDNA we obtained in previous studies. Cells were classified into several groups under different treatments. In the whole study, we made cell cultured on hard common cell culture plate remained very high stiffness, except results in **Figure 7**(two ECM stiffness 2D models, details in 2.2). We owned negative control (NC) (6 groups including HCC cells transfected with miR-3682-3p inhibitor NC in **Figure S1, S2, 2 , 4 and 7**, miR-3682-3p NC plasmids in **Figure 4**, sh-miR-NC in **Figure 3**, PHLDA1 NC plasmids in **Figure 5** and miR-3682-3p inhibitor NC/miR-3682-3p NC+PHLDA1 si-NC in **Figure 6**), miR-3682-3p (MHCC-97H and Hep3B transfected with miR-3682-3p mimics plasmid in **Figure 4**), miR-3682-3p inhibitor (MHCC-97H and SMMC-7721 cells transfected with miR-3682-3p inhibitor), PHLDA1 siRNA2 in MHCC-97H cells and miR-3682-3p inhibitor + PHLDA1 siRNA2 (MHCC-97H cells transfected with miR-3682-3p inhibitor + PHLDA1 siRNA2), PHLDA1(MHCC-97H transfected with PHLDA1 plasmid in **Figure 5**). The transfection was performed according to the instructions of lipofectamine 2000 (#11668019, Invitrogen, CA, USA). As for hypoxia culture, we kept MHCC-97H cell incubating in 5% CO2+1% O2 and 37°C surrounding for 12h and made it ready for further experiments. Three independent replicates were finished in each group.

### Apoptosis and flow cytometry

MHCC-97H cells and SMMC-7721 cells were both left different groups, and transfection performed was same as that in cell cycle experiments. All the cells were incubated with PE Annexin V in a buffer containing 7-Amino-Actinomycin (7-AAD) then analyzed by flow cytometry. Untreated cells were both primarily PE Annexin V and 7-AAD negative, indicating that they were viable and not undergoing apoptosis. There were primarily two populations of cells after 4 hours treatment: Cells that were PE Annexin V and 7-AAD negative, indicating that they were viable and not undergoing apoptosis; cells that were PE Annexin V positive and 7-AAD negative, indicating that they were undergoing apoptosis. There are also a minor population of cells were observed to be PE Annexin V and 7-AAD positive, indicating that they were in end stage apoptosis or already dead. All the treated cell samples were tested in flow cytometry (BD, #FACS Calibur, USA) and single PE negative/single 7-AAD negative and blank group were prepared for managed cell-selected windows. Three independent replicates were finished in each group.

### Reverse transcription quantitative polymerase chain reaction (RT-qPCR)

Levels of PHLDA1, miR-3682-3p and beta-actin were determined using RT-qPCR. The cells collected following transfection were dissolved for collecting the total RNA by a Trizol kit (Invitrogen Inc, CA, USA) ,whose RNA was reverse transcribed into cDNA(#k1622, ThermoFisher, Shanghai, China). The program was performed following Kits' protocols (for reverse transcription, 42℃ for 5 min, 70°C for 62 min and 4°C for standby; for qPCR, 95°C for 10 min, 95°C for 5 sec (cDNA)/95°C for 15 sec (miRNA RT product), 60°C for 30 sec and 72°C for 30 sec for 40 cycles within the last 3 steps). Beta-actin and U6 were used as internal references for mRNA and miRNA, respectively. The fold changes were calculated by means of relative quantification (2-ΔΔCT method). Four independent replicates were finished in each group.

### Dual luciferase reporter gene assay

A dual-luciferase-reporter-gene assay was established to determine that miR-3682-3p targeted PHLDA1. The 3'-untranslated region (3'-UTR) of the PHLDA1 gene was cloned into mi-vector (#E1330, Promega, WI, USA). Site-directed mutagenesis was performed on the potential binding sites of miR-3682-3p and the PHLDA1-mutant (MUT) vector or PHLDA1-wild (WT) were also arranged. The RL-vector containing Renilla luciferase (#E2241, Promega, WI, USA) was used as the internal control. The miR-3682-3p mimics or the NC plasmids were transfected into MHCC-97H and Hep3B cells together with PHLDA1-MUT or PHLDA1-WT. Two days after transfection, luciferase activity was detected in collected cells according to the protocol of the luciferase assay kit (GeneCopoeia, Maryland, USA). Three independent replicates were finished in each group.

### 5-ethynyl-2-deoxyuridine (EDU) assay

DNA replication was tested by using an EDULabeling/Detection Kit (#R11053.9, RiboBio, Guangzhou, China). Cells grown in 24-well plate at a density of 12×10^5^ cells/well were incubated with 325 μL EDU (50 μM) labeling media overnight at 37°C. After treatment with 4% paraformaldehyde and washing with 0.5% Triton X-100 and glycine/PBS several times, the plates were stained with Apollo working solution. Finally, DAPI was used to display cell nuclei. The proportion of EDU-positive cells was calculated after fluorescence microscopic analyses. Three independent replicates were finished in each group.

### Tumor formation model in nude mice

MHCC-97H cell suspension was prepared after digest by trypsin-EDTA (#HyClone, GE, Buckinghamshire, England). A mixture of PBS and Matrigel (#356234, BD, California, USA) at a ratio of 1:1 was prepared for suspending cells transfected with sh-NC and sh-miR-3682-3p at a final concentration of 4 × 10^6^ cells/150 μL.

A total of 16 male 4-week nude mice (SLAC Laboratory Animal Co. Ltd, Shanghai, China) were divided into four groups and included MHCC-97H and SMMC-7721 cell lines. Each cell subtype cell group was named as sh-NC group or sh-3682-3p group, in which had 4 mice respectively. Anesthetized by chloral hydrate, mice adaptive to new one-week-staying home in transplantation group were inoculated subcutaneously in the left posterior cervical subcutaneous sites respectively with prepared cells stored briefly in the 4 centigrade degree before. The mice were fed in the same specific pathogen-free environment, and were observed and measured weekly beginning from injections. Tumor length and width were recorded and the gross tumor volume was calculated according to the international common formula, volume = (length×width^2^)/2. On the 28th day, the mice ware sacrificed by chloral hydrate and tumors were removed. Three tumors in each transplantation group were weighed. All animal experiments were approved by the National Institutes of Health Guide for the Care and Use of Laboratory Animals.

### Immunohistochemistry (IHC stain)

The whole dyeing process consists of five contents: dewaxing, dyeing, dehydration, transparency and sealing. We chose 0.4 μM paraffin section of tumor nodules. Firstly, we finished a dewaxing protocol as common as any other stage in article such as Dou CW made (Gastroenterology 2018) 43. Then we made antigen retrieval step in Citrate Antigen Retrieval solution, which sections were heated in microwaves via steeping in boiling solution 20 min and then cooling in room temperature. After that, we followed the main IHC kit (#sp0001, SPlink Detection Kits, Zsbio, China) procedure including a). block endogenous peroxidase adding appropriate amount of endogenous peroxidase blocking agent, incubate at room temperature for 10 minutes; b). wash with PBS buffer for 3 minutes for 3 times; c). drop and seal the normal goat serum working fluid with 100 μL or appropriate amount of sealed normal goat serum working fluid, incubate at room temperature for 10 to 15 minutes, pour out the serum, do not wash; d) add 100μL or appropriate amount of primary antibody (rabbit polyclonal antibody to CASP8 (#ab220171, 1:400, Abcam, Cambridge, UK), rabbit polyclonal antibody to Ki-67 (#ab ab166667, 1:400, Abcam, Cambridge, UK)), and incubate at 4℃ for10 hours. Wash with PBS buffer for 3 minutes for 3 times; e). add 100μL or appropriate amount of biotin-labeled goat anti-rabbit IgG polymer and incubate it at room temperature for 10 to 15 minutes. Wash with PBS buffer for 3 minutes for 3 times; f). add 100 μL or appropriate amount of HRP-labeled streptomycin working liquid and incubate it at room temperature for 10-15 minutes. Wash with PBS buffer for 3 minutes for 3 times; g). add appropriate amount of freshly prepared DAB chromogenic solution and incubate at room temperature for 5 to 8 minutes; h). wash the tap water after dyeing, and incubate the hematoxylin staining solution for 20 seconds; Differentiation, rinse and return to blue; i). dehydration, transparency, tablet sealing; j). tablet reading. The staining results shall be observed and interpreted by a qualified pathologist under an optical microscope.

### Western blotting

A bicinchoninic acid (BCA) Protein Assay Kit (#WB003, FiveHeart, Xi'an, China) was used for measurement of the protein concentration after the total protein had been extracted in RIPA Lysis Buffer (#WB009, FiveHeart, Xi'an, China) . After separation with 12% sodium dodecyl sulphate-polyacrylamide gel electrophoresis (SDS-PAGE), the protein was transferred in liquid iced trans-buffer onto polyvinylidene fluoride (PVDF)(#IPVH00010, MerckMillipore, USA) membrane. the membrane blocked with 10% skimmed milk was incubated at 4°C for 12h with the addition of primary antibodies: mouse monoclonal antibody to β-actin (#ab6276, 1:5000, Abcam, Cambridge, UK), rabbit polyclonal antibody to PHLDA1(#ab133656, 1:2000, Abcam, Cambridge, UK), rabbit monoclonal antibody to FAS (#ab133619, 1:2500, Abcam, Cambridge, UK), rabbit monoclonal antibody to P21 (#ab188224, 1:2500, Abcam, Cambridge, UK) and rabbit monoclonal antibody to HIF-1a (#ab16066, 1:2500, Abcam, Cambridge, UK). After receiving three washes with Tris-buffered-saline supplemented with 0.05% Tween 20 (TBST), the membrane was incubated with goat anti-mouse IgG secondary antibody (#ab7072, 1:6000, Abcam, Cambridge, UK) or goat anti-rabbit IgG secondary antibody (#ab6721, 1:6000, Abcam Inc, Cambridge, UK) at room temperature for one and a half of hours. Next, the 0.45μm membrane was rinsed three times with PBST and exposed in western HRP substrate (#WBLUF0110, MerckMillipore, USA) under dark conditions. Relative protein levels of the target genes were expressed as the ratio of gray values of the target protein bands to those of the β-actin band. Three independent replicates were finished in each group.

### Tunel reaction fluorescein test

We chose paraffin section from samples in 2.9 and made any section complete and clean. Dewaxing step was equal as step in 2.10. Tissue sections after dewaxing can be pretreated in proteinase K (10 - 20 g/ml in 10 mM Tris/HCl, pH 7.4 - 8) the concentration, incubation time and temperature have to be optimized for each type of tissue. Following steps include Tunel reaction solution preparation and action. Green fluorescein shows the apoptosis cell and DAPI stain is ready for all cells in tissues. The steps above are references from In Situ Cell Death Detection Kit, Fluorescein (#11684795910, Roche, Philadelphia, USA). Three independent replicates were finished in each group.

### Transwell assays

The 8-μm Transwell chambers (Corning, USA) placed in 24-well plates were used for migration and invasion assays. In the invasion assays, the chamber was coated with a 70 μL mixture of Matrigel (#356234, BD, California, USA) and DMEM at a 1:6 ratio. Then, 200 μL of MHCC-97H transfected cells were seeded into the apical chambers, with complete medium added into the lower chamber. The chambers were then incubated at 37°C with 5% CO_2_. After a 36 h incubation, the cells were fixed and stained with 0.1-0.5% crystal violet. For the migration assay, the same procedure was followed omitting the Matrigel. The stained cells were counted under the microscope to determine the average number of cells in four randomly selected fields.

### Statistical analysis

The statistical analysis was conducted by using SPSS 19.0 (IBM. Armonk, USA) and GraphPad 6.0 (GraphPad Software, San Diego, USA). Mean ± standard deviation was statistics disposition for measurement data. Two-groups data were compared using t test and multiple-groups data were compared using one-way analysis of variance (ANOVA). Fischer's least significant difference (LSD) method was adopted for paired comparison. Enumeration data were presented as percentage and analyzed using chi-square test. A value of P<0.05, also appearance of P<0.01 or P<0.001, means statistical significance.

## Results

### HCC presented with high-expression of miR-3682-3p involving poor prognosis

**Figure 1:**
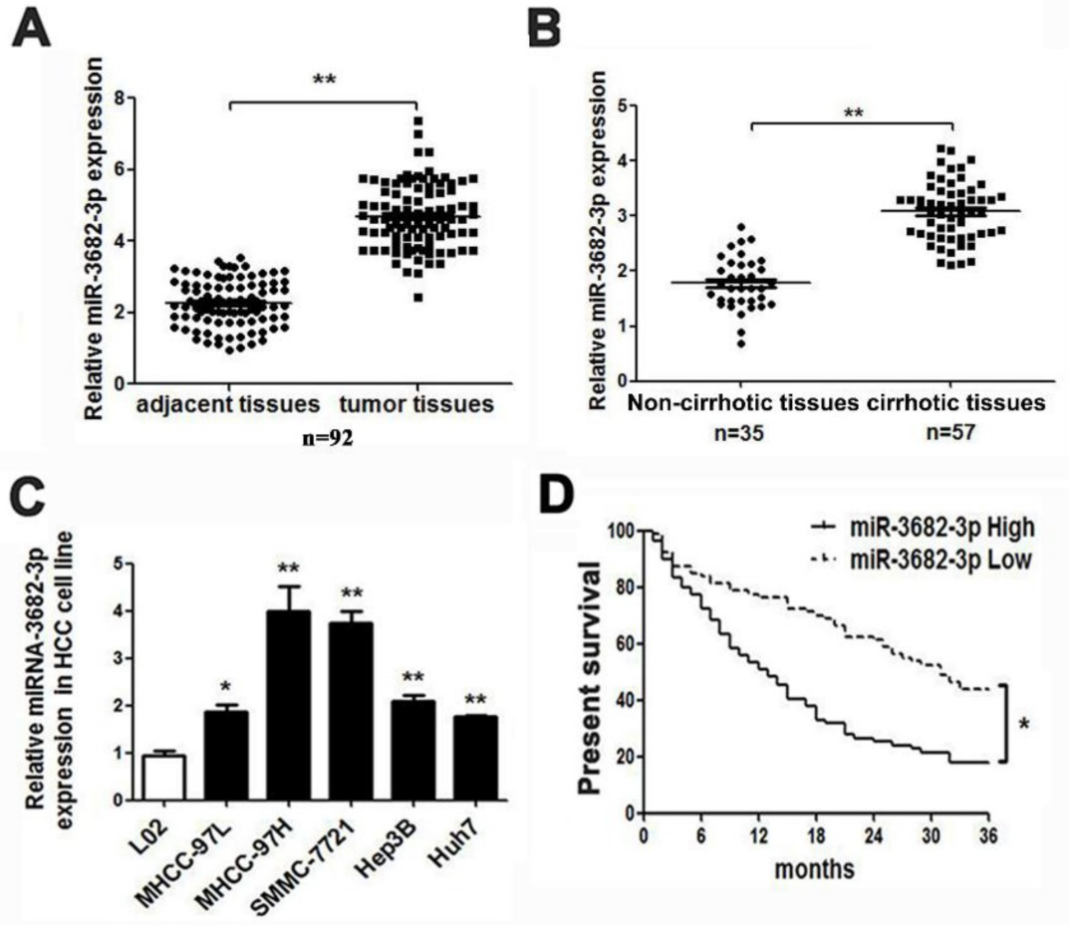
HCC presented with high-expression of miR-3682-3p involving poor prognosis. Comparing differences in the expression levels of miR-3682-3p between (A) HCC and adjacent tissues and (B) non-cirrhotic and cirrhotic tumor tissues. (C) HCC cell lines and the immortalized hepatic cell line LO2, U6 was used as internal control. (D) Overall survival was compared between HCC patients with high expression level of miR-3682-3p and those with low level of miR-3682-3p. *P < 0.05, **P < 0.01.

According to databases analysis, we found many miRNAs with abnormal expression in deceased or living samples (OncomiR: http://www.oncomir.org/). In HCC tissues, miR-3682-3p was in high level and related with poor prognosis, for the deceased specimens remained significant expression and up-regulation in 374 tumor samples and high-expression-related poor prognosis were obvious (**Figure S1B** from Starbase: http://starbase.sysu.edu.cn/ and OncomiR). Then, we tested the expression of liver cancer common cell lines and specimens in medical institution. The result of relative expression in HCC cell lines indicated that MHCC-97H and SMMC-7721 cell line with higher offensive showed bigger volume of miR-3682-3p. The expression of miR-3682-3p was higher not only in tumors, as expected, but also in cirrhotic samples, which contained 92 pairs of adjacent and liver tumor tissues and at least 35 sample with or without hepatic cirrhosis (**Figure 1A and 1B**). The absence of miR-3682-3p could be figure out in relative noninvasive cell lines as MHCC-97L and Hep3B, although the minimum of expression in any kind of cell line we chose remarkably exceeded the normal hepatic cells (**Figure 1C**). Considering the poor prognosis feature of HCC, we had obligation to illustrate the prognostic curve of different expression of such miRNA. In our institution, 80 and 79 samples with lower exhibition and higher expression of miR-3682-3p indicated a visible variation in prognosis, higher expression of miR-3682-3p viewed more inferior survival rate (**Figure 1D**). Besides, TCGA database corroborated the difference in surviving percentage of diverse volume of miR-3682-3p separated by median during 10 years (**Figure S1C**).

### Clinical significance of miR-3682-3p in HCC patients

To further confirm that expression of miR-3682-3p associated with the clinical features of HCC patients, we divided 92 HCC patients into high and low group according to median value of miR-3682-3p expression. We collected 46- low- expression group and 46-high -expression group for subsequent research. As shown in **Table 1**, we demonstrated that upregulated miR-3682-3p was significantly associated with tumor-node-metastasis (TNM) stage (III+IV), Edmondson stage (III+IV), cirrhosis features and multiple tumor nodes. These data suggest that miR-3682-3p is a potential biomarker for the clinical outcome of HCC patients.

### miR-3682-3p promotes proliferation and inhibits apoptosis of HCC

**Figure 2:**
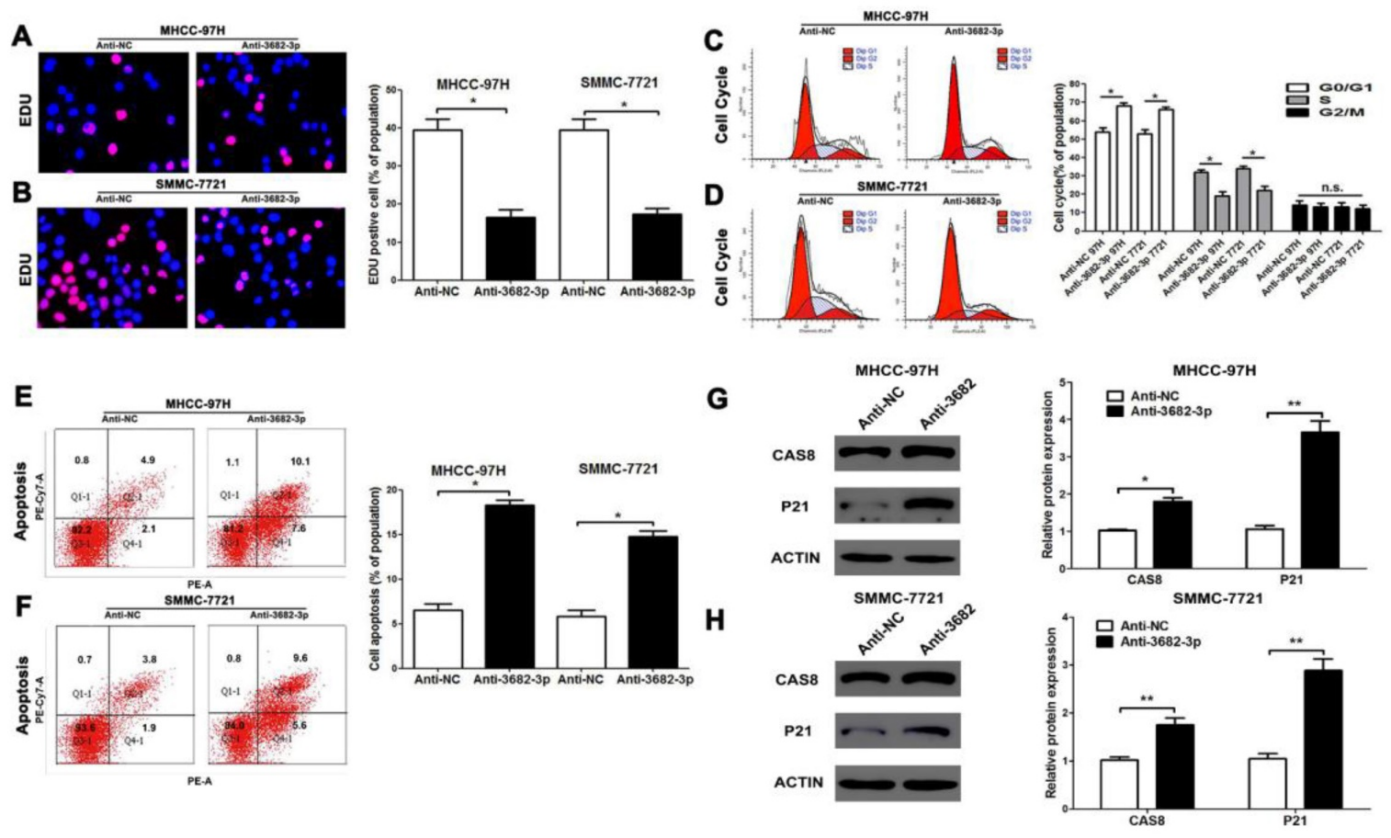
Down-regulated miR-3682-3p inhibits proliferation and promotes apoptosis of HCC in vitro. (A and B) EDU tests showed lower cellular vitality in both MHCC-97H and SMMC-7721 cell lines transfected with miR-3682-3p inhibitor. (C and D) Cell cycle were inhibited in G1 phase by down-expressing miR-3682-3p in MHCC-97H and SMMC-7721 cells. (E and F) The proportion of apoptosis in two cells transfected with miR-3682-3p inhibitor was lower than NC group. (G and H) The protein level of CAS8 and P21 were tested by WB. Downregulated miR-3682-3p promoted expression of CAS8 and P21. Bar figures indicated the statistics. n = 3 independent experiments. *P<0.05, n.s.= no significance.

Functional experiments were established to evaluate miR-3682-3p in HCC cell lines. MHCC-97H and SMMC-7721, both with high expression of miR-3682-3p were selected as cell lines for its knock-down (**Figure 1C**). For evidence suggested down-regulation was viable, further testes could be credible (**Figure S1D and S1E**). Comparing with negative control groups of two cell lines, cell EDU assay indicated that vitality of HCC cells diminished while miR-3682-3p has been down-regulated (**Figure 2A and 2B**). Similarly, complementary cell cycle test illustrated proliferation suppression in both cells (**Figure 2C and 2D**). The data demonstrated that higher expression of miR-3682-3p provided more capability of cell division. We evaluated another important behavior, apoptosis, by flow cytometry machine. Either MHCC-97H or SMMC-7721 demonstrated enhancement of apoptosis when miR-3682-3p high-expression was significantly eliminated (**Figure 2E and 2F**). The markers of apoptosis (caspase-8) and cell cycle (P21) had been tested by western blotting. The results showed the promotion of caspase-8 and P21 in group of anti-miR-3282-3p (**Figure 2G and 2H**). There showed the same feature that suppression of miR-3682-3p aggravated apoptosis and inhibited cell cycle in both cell lines. In addition, we tested the influence on migration and invasion of HCC cell transfected with miR-3682-3p inhibitor. However, nothing statistical significance was appeared in the results of MHCC-97H cell lines shown in Transwell (**Figure S2**). To sum up, higher expression of such miRNA supplied priority of cell division and apoptosis resistance, but has insignificant effects on migration and invasion.

### Knocking-down miR-3682-3p shows largest blocked growth of implanted tumor and acceleration of apoptosis in nude mice

**Figure 3:**
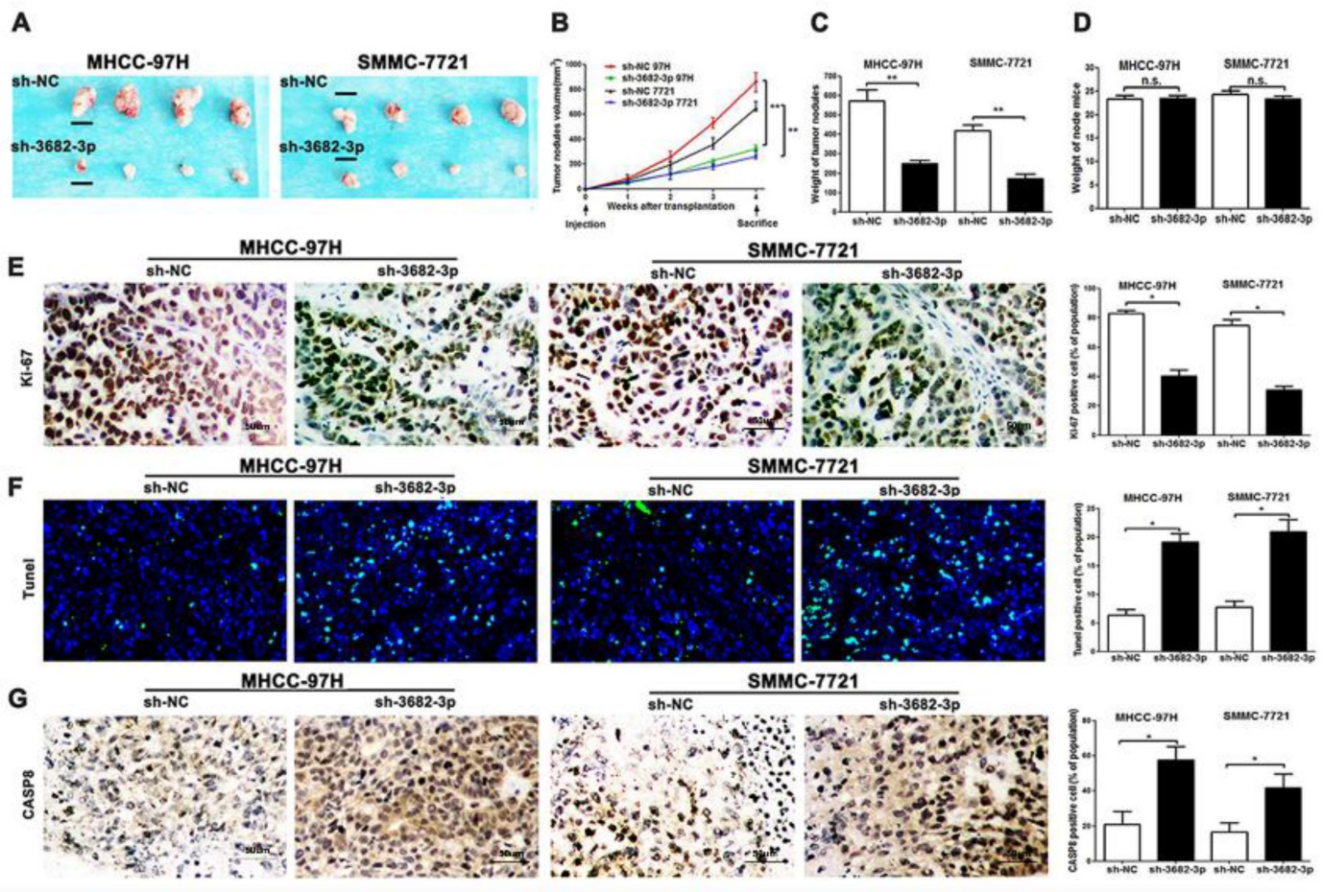
Down-regulated miR-3682-3p inhibits the nodules grow and promotes apoptosis in MHCC-97H and SMMC-7721 cells in nude mice and results of apoptosis markers. (A) Nodules comparison figure in sh-miR-3682-3p and sh-NC group. (B) Week-measurement curves of nodules in each group. (C and D) Tumor weight and mice weight before sacrifice. (E, F and G) Representative IHC staining or fluorescein of nodules sections of Ki-67, Tunel and CASP8 in sh-miR-3682-3p and sh-NC cells. Bar figures indicate the statistics of number of lung nodules. Bar figures indicated the statistics. *P<0.05, **P<0.01. n= 3 independent experiments.

In vitro, we have made confirmation about the oncogenesis of miR-3682-3p. In vivo, to confirm the effects of miR-3682-3p, we used subcutaneous tumor transplantation models to be the reflection of tumor oncogenesis. The group of miR-3682-3p down-expression, verified by post-transfection sh-miR-3682-3p qPCR, illustrated weakened capacity of HCC xenografts (**Figure S1F**). Continuing measurement of injected tumor nodules suggested significant suppression of tumor volume in sh-miR-3682-3p group (**Figure 3A**). According to the weekly measurement of tumor nodes, we could clarify the disappearance of divergence of sh-3682 group in (**Figure 3B**). The weight of nodules showed that tumor sites in negative group were bigger than sh-group via no statistical difference of mice weights, genders and ages (**Figure 3C and 3D**). Then we tested proliferation and apoptotic markers in tissues level. IHC level of Ki-67 and caspase-8(CASP8), and Tunel tests in fluorescein level indicated the statistics of nodules growth and ratio of apoptotic cells in sections. The data suggested that knocking down miR-3682-3p could weaken tumor proliferation and promoted apoptosis in tumor nodes of MHCC-97H and SMMC-7721 cell lines (**Figure 3E, 3F and 3G**). Taken together, these results suggest that miR-3682-3p promotes oncogenesis and suppresses apoptosis behaviors of HCC in vivo.

### PHLDA1 is a target of miR-3682-3p

**Figure 4:**
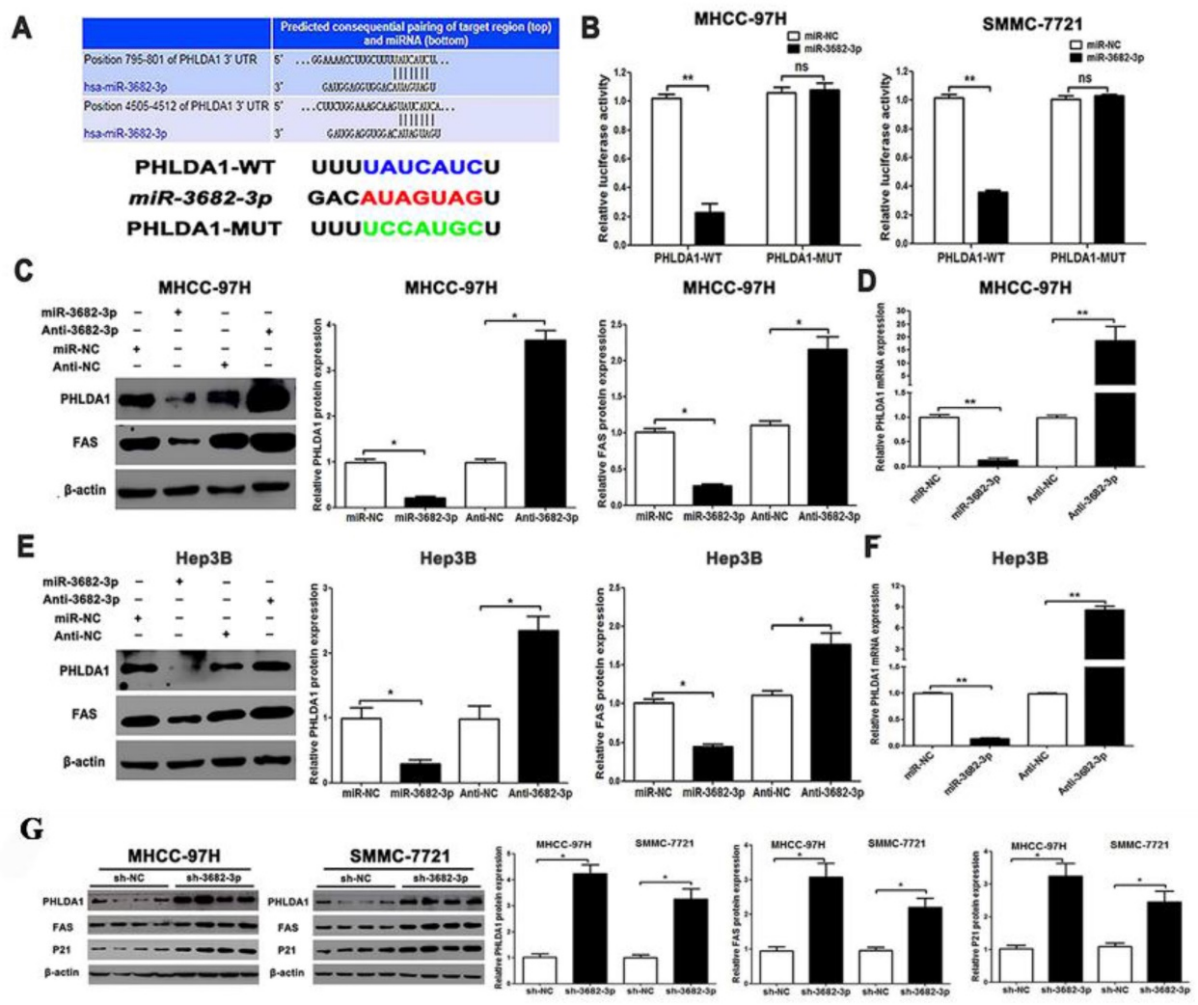
PHLDA1 is a direct target of miR-3682-3p in HCC cells. (A) TargetScan database shows miR-3682-3p putative binding sequence in the 3'-UTR of PHLDA1. The mutant binding site is generated in the complementary site for the seed region of miR-3682-3p. Relation of miR-3682-3p and PHLDA1 in HCC in Starbase database. (B) miR-3682-3p significantly suppressed the luciferase activity that carried wild-type (WT) but not mutant (MT) 3'-UTR of PHLDA1, which led to a notable increase in the luciferase activity of WT 3'-UTR of PHLDA1 in MHCC-97H and Hep3B cell lines. (C) Protein expression of PHLDA1 and Fas, and (D) qRT-PCR analysis of PHLDA1 mRNA expression in MHCC-97H cells with miR-3682-3p inhibitor or NC transfection in MHCC-97H. (E) Protein expression of PHLDA1 and Fas, and (F) qRT-PCR analysis of PHLDA1 mRNA expression in Hep3B cells transfected with miR-3682-3p inhibitor or mimics. (G) WB results of PHLDA1, Fas and P21 relative volume in nodule tissues. Bar figures indicated the statistics. *P<0.05, **P<0.01. n = 3 independent experiments.

Since PHLDA1 was predicted to be a target of miR-3682-3p by three databases including Starbase v3.0, TargetScan v7.2 (http://www.targetscan.org/vert_72/) and MiRgator v3.0 (http://mirgator.kobic.re.kr/) (**Figure 4A, Figure S3A and S3B**), dual-luciferase reporter gene assay was performed to verify the relationship between miR3682-3p and PHLDA1. To make confirmation of statistic reliability and consistency, we chose MHCC-97H and Hep3B cell lines to finish further tests, which indicated reverse expression level of miR-3682-3p. Compared with the miR-NC group, there was a clear fall in the luciferase activity of WT-PHLDA1 in the miR-3682-3p mimic transfected group in each cell lines (**Figure 4B**). Besides, there was nothing statistically different in the luciferase activity of the MUT-PHLDA1 group. About the mRNA level of PHLDA1 and protein level of PHLDA1 and Fas, there were converse changes in over- or down-expression of miR-3682-3p in MHCC-97H cell line (**Figure 4C and 4D**). Interestingly, the same changing trend of PHLDA1 and Fas protein and mRNA level had been demonstrated by western blotting and qPCR in Hep3B group, whose shortening of these targets was statistically significant (**Figure 4E and 4F**). To explore the pathway that miR-3682-3p effected on division and apoptosis, we examined protein level of PHLDA1, Fas (or called TNF receptor superfamily member 6) and cyclin dependent kinase inhibitor 1A (CDKN1A, also known as P21) in vivo by testing transplanted tumor nodules. These three protein showed higher expression in sh-3682-3p group tissues, which illustrated the activation of PHLDA1-Fas pathway and inhibition of cell cycle (**Figure 4G**). In addition, we collected the data of RNA expression of PHLDA1 and miR-3682-3p in 92 HCC samples. Correlation analysis was used to confirm that the relationship between this miRNA and the downstream gene. We found the statistical significant negative correlation between miR-3682-3p and PHLDA1 (**Figure S3C**). We also tested the relationship between miR-3682-3p and Fas, and PHLDA1 and Fas in database (Starbase v3.0 and Cbioportal v3.04, **Figure S3D and S3E**). Therefore, with the support of results in vitro and in vivo, miR-3682-5p could specifically bind to PHLDA1 gene.

### PHLDA1 plays a role in HCC inhibitor and accelerates apoptosis

**Figure 5:**
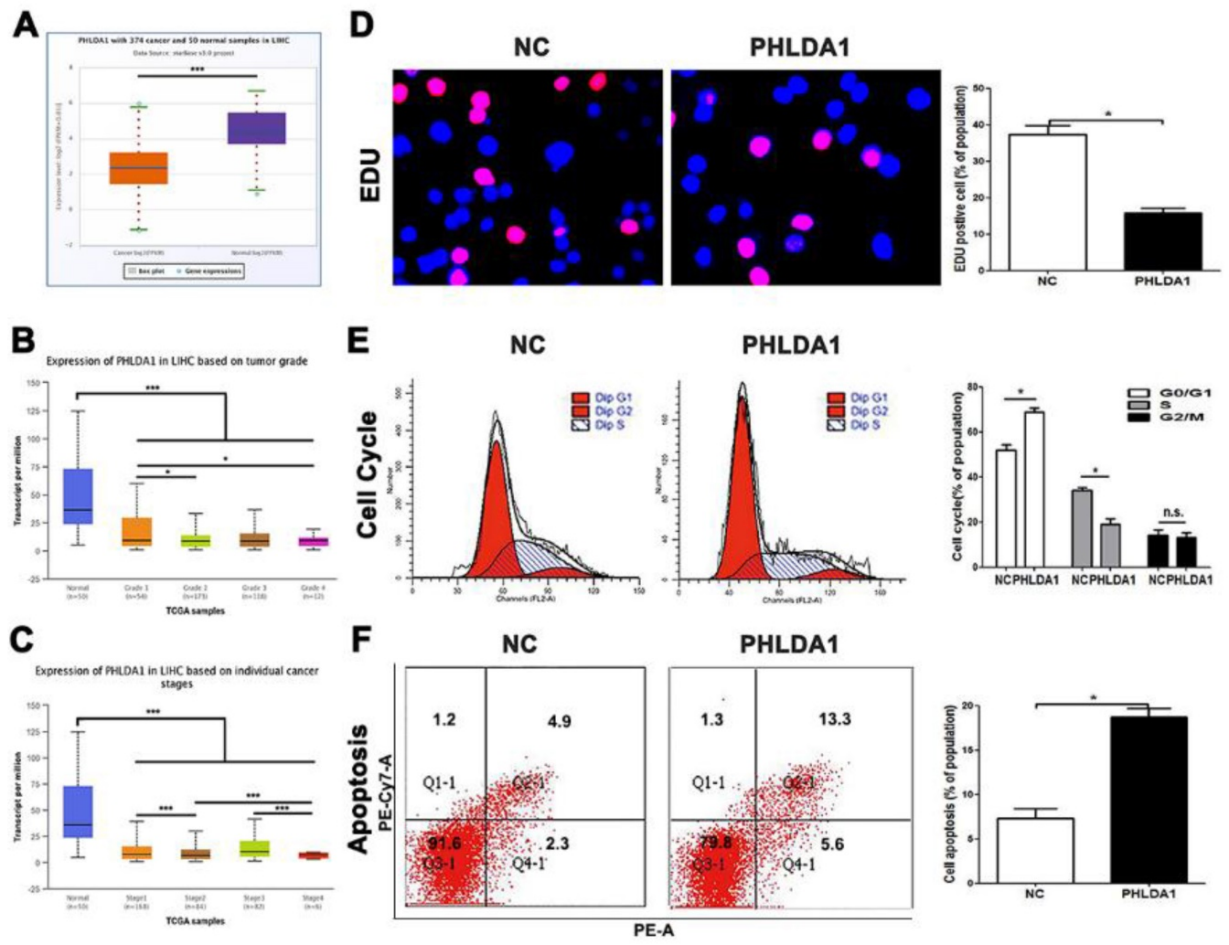
PHLDA1 was downregulated in HCC and regulated the cell growth and apoptosis in HCC. (A) PHLDA1 expression in HCC tissues in Starbase. (B and C) PHLDA1 expression in different HCC stages and grades in TCGA database. (D) EDU tests showed lower cellular vitality in MHCC-97H cell lines transfected with pcDNA-PHLDA1. (E) The proportion of G1 phase in cell cycle phases in MHCC-97H cells transfected with pcDNA-PHLDA1 was higher than NC group. (F) Cell apoptosis was promoted by overexpressed PHLDA1 in MHCC-97H cells. Bar figures indicated the statistics. *P<0.05, **P<0.01. n.s.= no significance. n = 3 independent experiments.

Though we have claimed before, the researches on functions of PHLDA1 in HCC was poorly reported, several testes have showed multiple potentials of PHLDA1 in other cancers, especially the promotion of apoptosis. However, there was no final conclusion that PHLDA1 played a positive or negative status in HCC, for inconsistent researches were published during the 10 years, which discussed the multiple function of PHLDA1 in same kind of tumor. According to database, we had understood that PHLDA1 was low-expressed in HCC tissues and related to tumor grades and differentiation (**Figure 5A, 5B and 5C**). The qPCR testing PHLDA1 mRNA expression in HCC cell lines, MHCC-97H cell line, led us to select lowest expression of PHLDA1 to finish examinations (**Figure S4A**). Our results came from functional experiments by HCC cells over-expressed PHLDA1 (**Figure S4B**). Cell cycle and EDU and the ratio of cells of apoptosis gave the inhibition evidence of proliferation behaviors and enhancement of apoptosis mentioned before in PHLDA1-up-regulated group (**Figure 5D, 5E and 5F**). In short, PHLDA1 plays a role of inhibitor in liver cancer, which inhibited cell division and accelerate apoptosis.

### Up-regulation of miR-3682-3p or silencing of PHLDA1 enhances HCC cell proliferation and suppresses apoptosis

**Figure 6:**
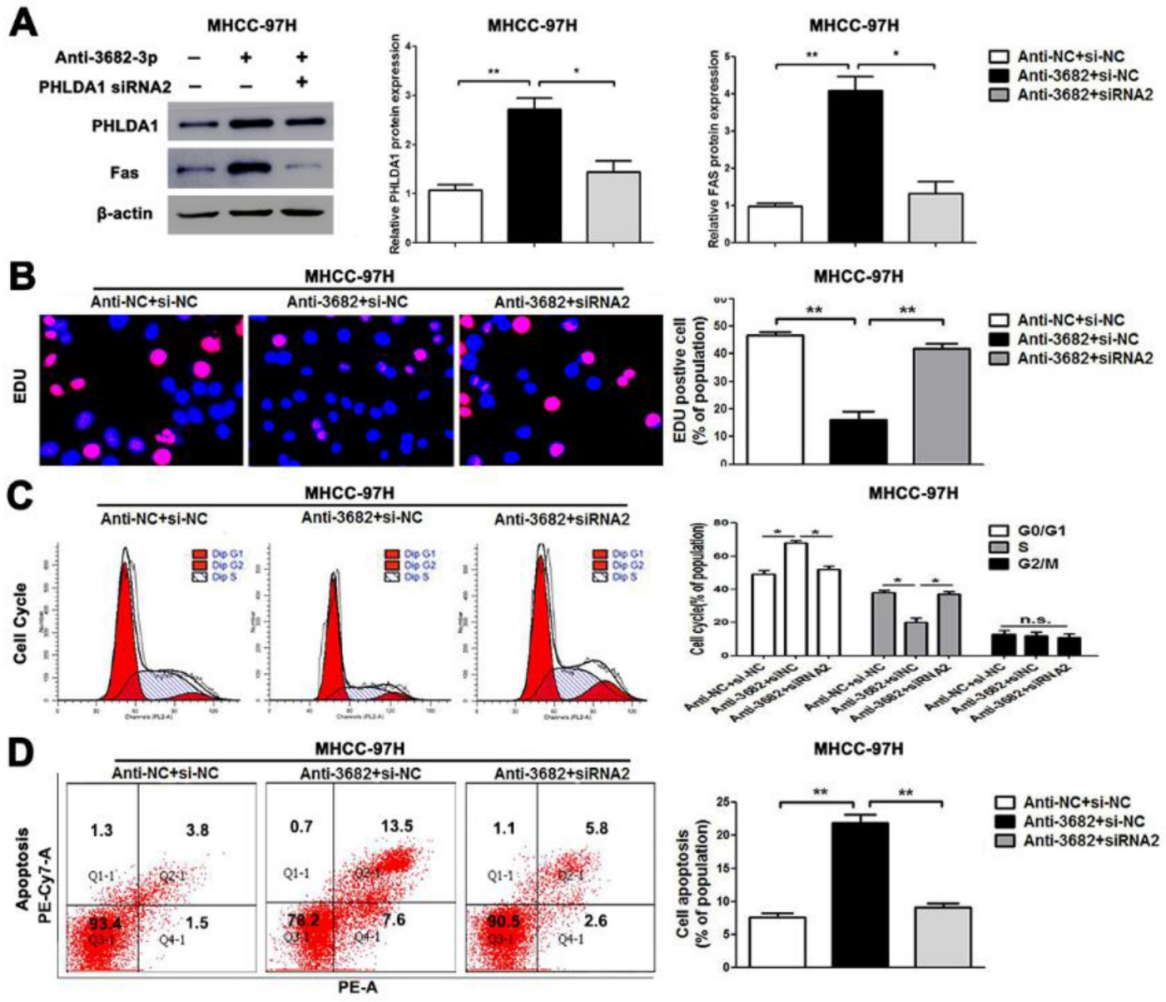
Alterations of PHLDA1 partially abolish miR-3682-3p-mediated HCC cell proliferation and apoptosis in MHCC-97H cells. (A) PHLDA1 and Fas protein levels were tested by WB in miR-3682-3p-down-expressed MHCC-97H cells or cells transfected with inhibitor and PHLDA1 siRNA. (B) EDU tests showed lower cellular vitality in MHCC-97H cell lines transfected with miR-3682-3p inhibitor. PHLDA1 knockdown abrogated the effects of miR-3682-3p knockdown in MHCC-97H cells. (C) The proportion of cell cycle G1 phases in MHCC-97H cells transfected with miR-3682-3p inhibitor was higher than NC group. PHLDA1 knockdown abrogated the effects of miR-3682-3p knockdown in MHCC-97H cells. (D) Cell apoptosis was promoted by down-expression of miR-3682-3p in MHCC-97H cells. PHLDA1 knockdown abrogated the effects of miR-3682-3p knockdown in MHCC-97H cells. Bar figures indicated the statistics. *P<0.05, **P<0.01. n.s.= no significance. n=3 independent experiments.

Next, comparing with the NC groups, a significant decrease in PHLDA1 and Fas protein level in the miR-3682-3p inhibitor and a rescue in miR-3682-3p inhibitor + si-PHLDA1 groups (**Figure 6A**) was flashed in MHCC-97H cells, while siRNA efficiency was tested primarily and helped us make decision of siRNA2(**Figure S4C**). Moreover, when we compared with the NC groups, we could figure out that cell vitality confirmed by EDU and cell cycle in the anti-miR-3682-3p group was diminished, at the same time, there was a significant difference detected in the miR-3682-3p inhibitor + si-PHLDA1-2 group (**Figure 6B and 6C**). The reverse trend flashed in apoptosis assay and the influence of anti-miR-3682-3p and silencing PHLDA1 simultaneously in apoptotic motions of MHCC-97H cell line (**Figure 6D**). The copies of experiments were managed in Hep3B cell, which gave similar results of cell growth and apoptosis (**Figure S5A-E**). These findings suggested that the miR-3682-3p could promote HCC carcinogenesis while inhibiting the PHLDA1-Fas signaling.

### miR-3682-3p up-regulation is enhanced in high ECM stiffness

**Figure 7:**
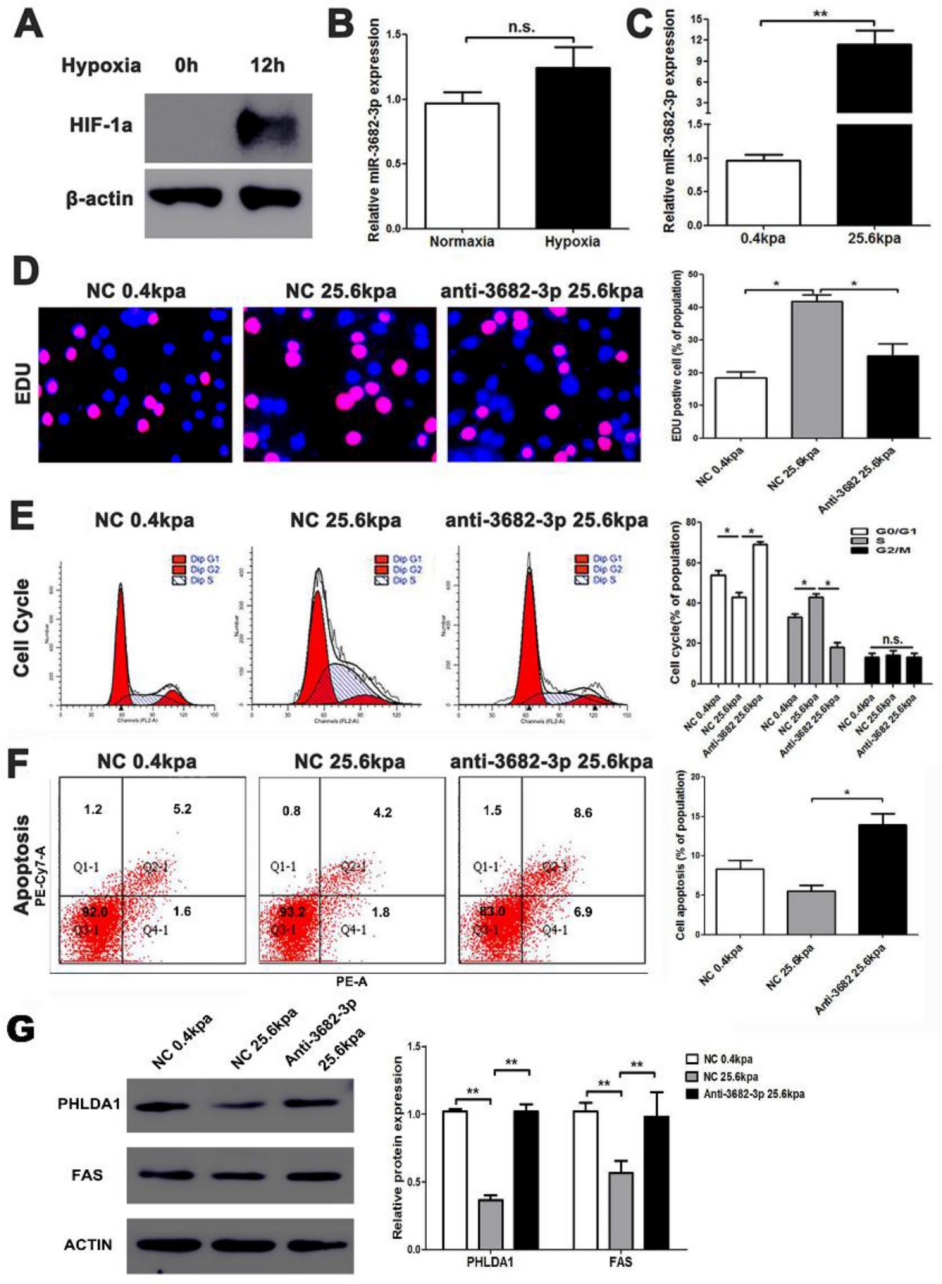
High ECM stiffness up-regulates miR-3682-3p expression. (A) Hypoxic condition was tested by western blotting for HIF-1a expression, and (B) The expression of miR-3682-3p in hypoxia or normoxia condition. (C) The expression of miR-3682-3p in high or low miR-3682-3p expression in MHCC-97H cell lines. (D) EDU shows 25.6kpa stiffness that resulted in larger miR-3682-3p expression demonstrated higher cell viability. While whittling down miR-3682-3p, the significant cellular viability on 25.6 kpa anti-3682-3p group changed reversely. (E) Cell cycle tests show 25.6kpa stiffness that resulted in larger miR-3682-3p expression demonstrated higher cell division. While whittling down miR-3682-3p, the significant cellular division on 25.6kpa anti-3682-3p group changed reversely. (F) Apoptosis in flow cytometry shows 25.6kpa stiffness that resulted in larger miR-3682-3p expression demonstrated low ratio of apoptosis. While whittling down miR-3682-3p, the low apoptosis on 25.6kpa anti-3682-3p group changed reversely. (G) Protein level of PHLDA1 and Fas in 25.6 kpa group were lower than that in 0.4kpa group. While whittling down miR-3682-3p, the expression of the two proteins was promoted again. Bar figures indicated the statistics. *P<0.05, **P<0.01. n.s.= no significance. n=3 independent experiments.

**Figure 8:**
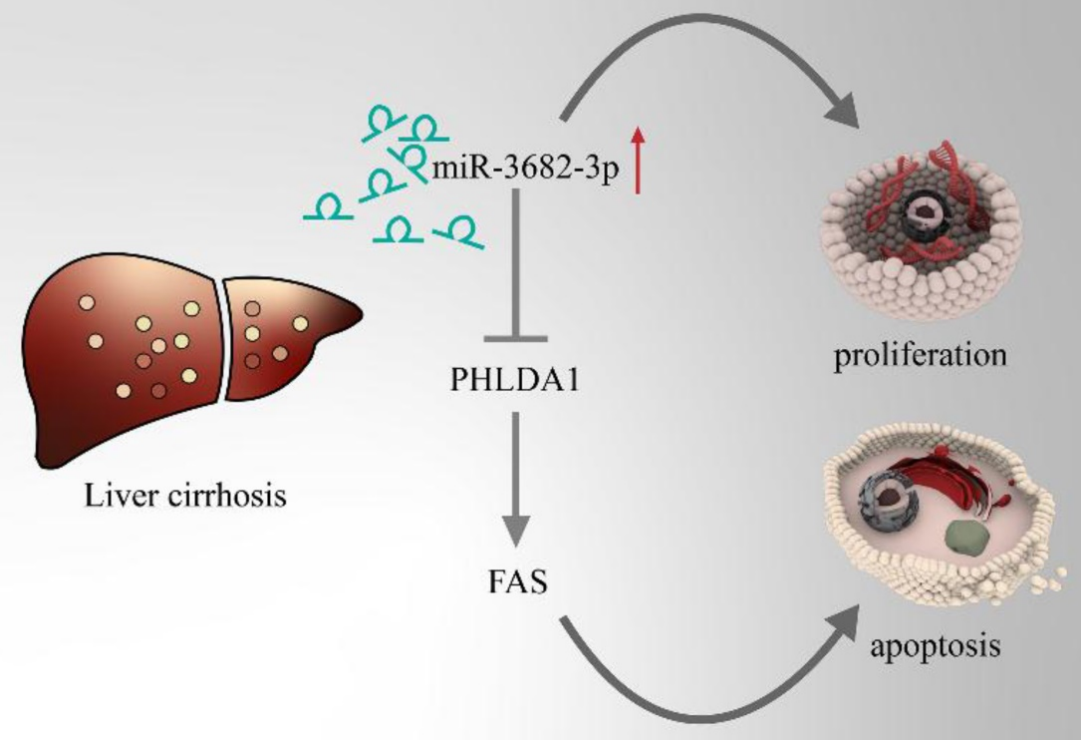
Model pattern of high-ECM-stiffness-miR-3682-3p-PHLDA1-Fas pathway.

HCC, one of the famous liver-fibrosis-related cancer, exhibited higher aggressive features under regulation of high ECM stiffness. Reports of ECM-stiffness-mediating miRNAs in liver cancer remain expected and meaningful. In our previous surveys, we focused on hypoxia and hepatic stellate cells (HSCs). To find out the underlying mechanism of high-expression of miR-3682-3p, we firstly tried to test the relationship between hypoxia and miR-3682-3p. However, any statistical significance of the expression of this miRNA could not be figured out in hypoxic culture condition (**Figure 7A and 7B**). Our microarray of activated HSCs showed several up-regulated gene of ECM remodeling markers like Secreted Protein Acidic And Cysteine Rich (SPARC), metalloproteinase 10 (MMP10), Collagen Type V Alpha 1 Chain (COL5A1), Collagen Type VII Alpha 1 Chain (COL7A1) and Collagen Type XVI Alpha 1 Chain (COL16A1), some of these markers illustrated strong relationship to miR-3682-3p(data not shown). Then we tested the expression of miR-3682-3p via low and high ECM stiffness. It was obvious that miR-3682-3p could be promoted significantly on 25.6kpa stiffness (**Figure 7C**). Cell functional experiments established by using MHCC-97H cell lines (EDU, cell cycle and apoptosis) supported the hypothesis that high ECM stiffness promoted miR-3682-3p expression. The 25.6kpa stiffness that resulted in larger miR-3682-3p expression demonstrated higher cell viability and low ratio of apoptosis. While we whittled down miR-3682-3p, the significant cellular viability and apoptosis on 25.6kpa anti-3682-3p group changed reversely (**Figure 7D, 7E and 7F**). In addition, we tested the protein level of PHLDA1-Fas axis and the results supported our previous prediction (**Figure 7G**). Therefore, we indicated that miR-3682-3p up-regulation was enhanced in high ECM stiffness and whole model of this pathway was shown in **Figure 8**.

## Discussion

Large numbers of miRNAs have been reported to modulate pathological processes of malignancies, such as differentiation, apoptosis and remodeling of cytoskeleton, even migration, invasion of cancer cells. At the beginning of this study, we noted that there was an apparent abnormal overexpression of miR-3682-3p in HCC, while PHLDA1 expression was very low in HCC tissues. With the support of databases, we identified a poor prognosis in the high-miR-3682-3p group, which was associated with several malignant characteristics, such as tumor-node-metastasis (TNM) stage (III+IV), Edmondson stage (III+IV), cirrhosis features and multiple tumor nodes. Not only the previous predication of relation between high ECM stiffness and miRNA, but also the examination of expression of miR-3682-3p in cirrhosis samples or higher stiffness made the confirmation of oncogenesis and upregulation of such miRNA under great pressure of rigidity. Although there has been no prior research on this miRNA, we could easily explore researches into other miRNAs and HCC growth and apoptosis progression via high ECM stiffness 44-47. Moreover, there is a downexpression of PHLDA1 in HCC, which could be considered as an underlying bio-marker for the treatment of HCC 42.

miRNAs play an independent role in the pathogenesis and progress of almost any kind of cancers. As for solid cancers, breast cancer popularity in high ECM ranks the first and increasing articles about matrix have shown several key proteins mediate ECM modeling, which are responsible for their modulations in cellar differentiation, as well as complex relevance mainly about transcribing and suppressing directly down-stream targets^8^. As frequent as liver cirrhosis appearance, HCC promoted on high ECM stiffness exerts a terrible influence on human bodies. The novel miRNA, miR-3682-3p, has never been reported in any article of neoplasms including HCC. With the supports of advanced analysis of considerable amount of HCC samples, we fund the new miR-3682-3p and devoted to reveal the functions of such miRNA and further tested the high-ECM-stiffness-regulated axis in HCC.

According to recent studies, in HCC, ECM modification and remodeling mechanism have been tested and the research showed that many significant genes mediate ECM modification and remodeling, such as HBV-x protein, which could remodel ECM through HIF-1α/LOX pathway to promote HCC metastasis 49. Activated hepatic stellate cells drive fibrogenesis, changing of the matrix quantity and stiffness 9. Increased stiffness provided a reservoir for bound growth factors. In addition, previous article showed ECM accumulation was followed by enhanced expression of cancer-stem-cell-markers, including clusters of CD44, CD133, cysteine-X-cysteine receptor 4 (CXCR4), and NANOG 7. Scientists who are major in biomedical engineering indicated that “cells in deformable matrices stretched matrix fibers to store elastic energy; subsequent adhesion failure triggered sudden matrix recoil and rapid cell translocation 6, 48.” From now on, we have several answers about how collagen, actin and actomyosin regulate the movement of cancer cells and many interstitial cell secreted cellular factors accelerate ECM formation. Increasing matrix stiffness promotes proliferation, migration and chemotherapeutic resistance, whereas a comfortable environment for reversible cell-dormancy and stem cell characteristics in HCC 8.

Proliferation and apoptosis are the two main aggressive behaviors of any kind of solid tumor, especially HCC. Researchers on causes and signals about two features gradually complete. Increasing numbers of studies focused on the tumor microenvironment and the cancer-promoting effect of high ECM stiffness. High ECM stiffness is a common characteristic of solid tumors such as pancreatic cancer, HCC and breast cancer 8, 45, 49, 50. ECM remodeling plays an irreplaceable role in tumor heterogeneity, which causes abnormal gene expression and protein secretion in one tumor nodules. ECM environment is closely related to metastasis, apoptosis and invasions respectively 6-8, therefore, identifying pathways and down-streams of high ECM stiffness conditions might lead to therapeutic candidates of HCC. Angiogenesis, reprogramming for cytoskeleton, extracellular matrix remodeling and promotion of inflationary cell chemotaxis turn out to be consequences of two or three dimensional high ECM stiffness 8, 9. In our study, like normal test in hypoxia researches, we declaimed miR-3682-3p was over-expressed in high ECM stiffness condition, which led to promotion of carcinogenesis. We have indicated the relationship between miR-3682-3p and ECM stiffness, but further mechanism about how high ECM stiffness regulates expression of miR-3682-3p is still unknown. We are preparing the systemic exploration of direct or indirect evidence that may prove high ECM stiffness up-regulated miR-3682-3p.

According to prediction results, pri- or pre-miR-3682-3p sequence is provisional, so we still have no effective progress of primary miR-3682-3p promoter information. However, we unless know about miR-3682-3p is up-regulated and whether caused by long none-coding or circular RNA is under exploration. PHLDA1 is a well-known gene that leads to Fas expression and has been reported to be relative for apoptosis and autophagy in plenty of high-quality reports. According to the fact that PHLDA1 accelerate the cell death of HCC, we may make a bold hypothesis that relative high ECM stiffness condition in HCC could directly weaken PHLDA1-induced apoptosis. At least, such presumption is based on mesenchymal stem cell differentiation on high ECM stiffness and regulation via many other pathways.

In conclusion, the aforementioned results noted that miR-3682-3p causes the indulgence of cell proliferation and inhibition of apoptosis in HCC through the direct downregulating PHLDA1 via high ECM stiffness condition. However, this study remains at the period before clinical application. The mechanism of 2D ECM stiffness and direct protein or gene regulates miR-3682-3p have not been elucidated. Therefore, further studies of direct regulation between high ECM stiffness and miRNAs or PHLDA1 need to be broaden and we will expound more upon the function of miR-3682-3p in different tumor microenvironment.

## Supplementary Material

Supplementary figures.

## Figures and Tables

**Table 1:** Correlation between the clinicopathologic characteristics and miR-3682-3p expression in HCC (n = 92).

Clinical parameters	Cases	Expression level		*P* value
		MiR-3682-3p^high^ (n=46)	MiR-3682-3p^low^ (n=46)	
Age(years)				
<50 years	33	21	12	0.813
≥50 years	59	25	34	
Gender				
Male	76	37	39	0.788
Female	16	9	7	
Tumor size (cm)				
<5cm	36	19	17	0.831
≥5cm	56	27	29	
Tumor number				
solitary	66	28	38	0.036*
multiple	26	18	8	
Edmondson				
Ⅰ+Ⅱ	39	11	28	0.002**
Ⅲ+Ⅳ	53	35	18	
TNM stage				
Ⅰ+Ⅱ	48	16	32	0.002**
Ⅲ+Ⅳ	44	30	14	
Venous invasion				
Present	52	31	21	0.06
Absent	40	15	25	
AFP				
<40ng/ml	24	7	17	0.314
≥40ng/ml	68	39	29	
Cirrhosis				
positive	57	35	22	0.01**
negative	35	11	24	

HCC, hepatocellular carcinoma; AFP, alpha-fetoprotein; TNM, tumor-node-metastasis. *Statistically significant.

## Abbreviations

HCC: hepatocellular carcinoma
miRNAs: microRNAs
IHC: immunohistochemistry
ANOVA: one way analysis of variance
ECM: extracellular matrix stiffness
PHLDA1: Pleckstrin Homology Like Domain Family A Member 1
ER: endoplasmic reticulum
7-AAD: 7-Amino-Actinomycin
RT-qPCR: Reverse transcription quantitative polymerase chain reaction
TNM: tumor-node-metastasis
