# Supplementary material for: High-matrix-stiffness induces promotion of hepatocellular carcinoma proliferation and suppression of apoptosis via miR-3682-3p-PHLDA1-FAS pathway
Source: J Cancer. 2020 Aug 25;11(21):6188–203. doi: 10.7150/jca.45998 (PMC7532500; doi:10.7150/jca.45998)

Supporting Figure Legend

Figure 1. some key steps of methods. (A) ingredient of each ECM stiffness gel. (B) miR-3682-3p expression in Starbase and OncomiR databases (relation to survival rate). (C) miR-3682-3p prognosis in TCGA database (374 HCC patients). (D and E) Transfection efficiency of plasmid and inhibitor in MHCC-97H and SMMC-7721 cell lines. (F) Knocking-down efficiency of sh-3682-3p was viable for mice experiments.

Figure 2. miR-3682-3p had insignificant influence on HCC cells migration and invasion. n.s.= no significance. n=3 independent experiments.

Figure 3. Some relative expression of target gene in our study. (A) miR-3682-3p relative genes lists followed by correlation index in descending order in HCC tissues(green color means the negative relation). (B) relation of miR-3682-3p expression and PHLDA1 expression in HCC samples in Starbase. (C). relationship between miR-3682-3p and PHLDA1 in HCC samples from 92 patients. (D) relation of miR-3682-3p expression and Fas expression in HCC samples in Starbase. (E) relation of Fas expression and PHLDA1 expression in HCC samples in cBioportal database.

Figure 4. PHLDA1 mRNA expression in different treatments. (A) PHLDA1 mRNA level in HCC cell lines. (B) Ove-regulation efficiency of pcDNA-PHLDA1 plasmids or (C) Down-regulation efficiency of PHLDA1 siRNA. *P < 0.05, **P < 0.01.

Figure 5. Alterations of PHLDA1 partially abolish miR-3682-3p-mediated HCC cell proliferation and apoptosis in Hep3B cells. (A and B) miR-3682-3p-down-expressing Hep3B cells that were transfected with inhibitor or PHLDA1 expression siRNA were subjected to western blot analysis for PHLDA1 and Fas. (C) EDU tests showed lower cellular vitality in Hep3B cell lines transfected with miR-3682-3p inhibitor. (D) PHLDA1 knockdown abrogated the effects of miR-3682-3p knockdown in HEP3B cells. The proportion of cell cycle phases in Hep3B cells transfected with miR-3682-3p inhibitor was lower than NC group. PHLDA1 knockdown abrogated the effects of miR-3682-3p knockdown in Hep3B cells. (E) Cell apoptosis was promoted by down-expression of miR-3682-3p in Hep3B cells. PHLDA1 knockdown abrogated the effects of miR-3682-3p knockdown in Hep3B cells. *P<0.05, **P<0.01. n.s.= no

significance. n=3 independent experiments.

Supporting Figure 1

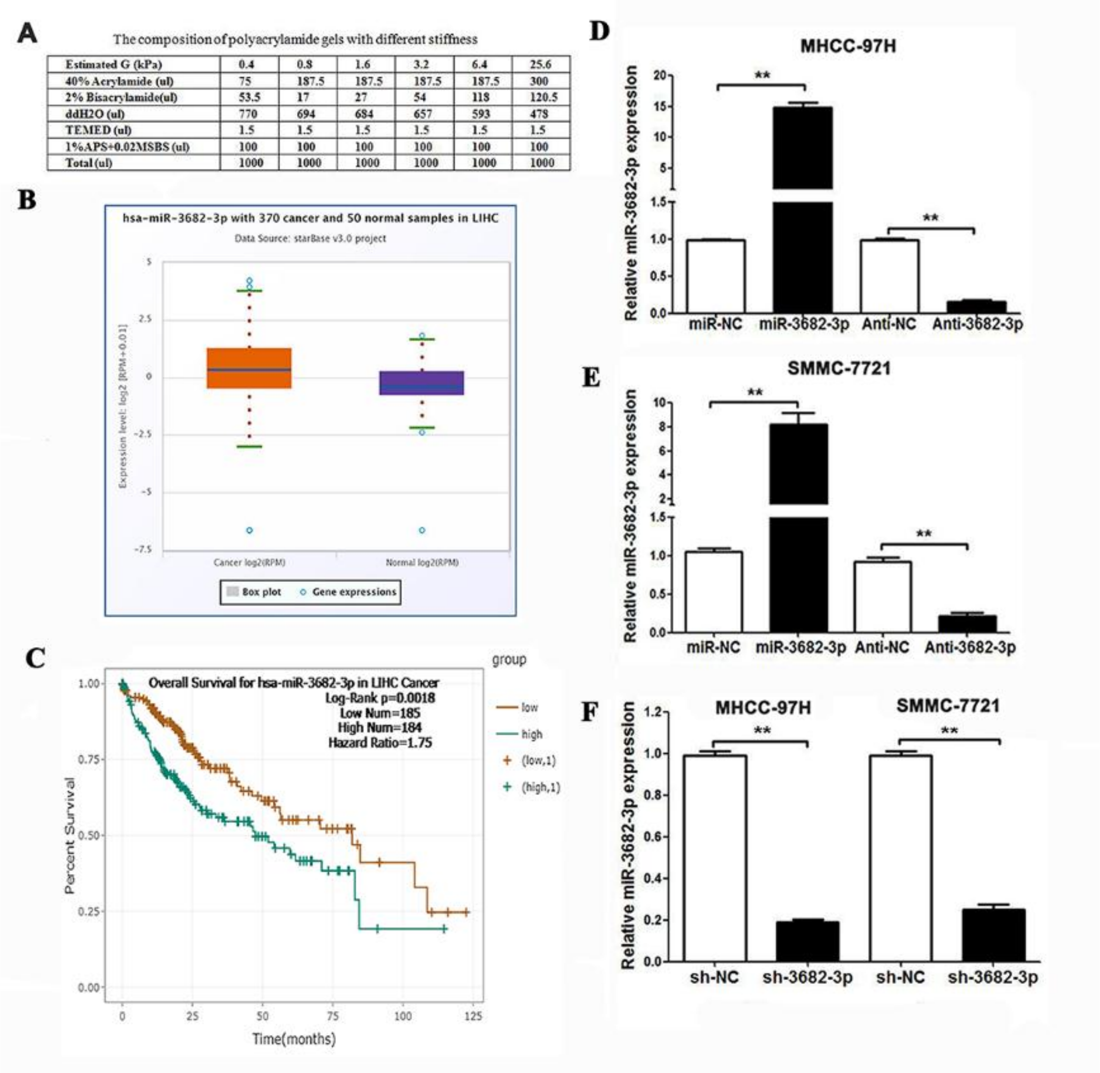

A

The composition of polyacrylamide gels with different stiffness

Estimated G (kPa)	0.4	0.8	1.6	3.2	6.4	25.6
40% Acrylamide (ul)	75	187.5	187.5	187.5	187.5	300
2% Bisacrylamide(ul)	53.5	17	27	54	118	120.5
ddH2O (ul)	770	694	684	657	593	478
TEMED (ul)	1.5	1.5	1.5	1.5	1.5	1.5
1%APS+0.02MSBS (ul)	100	100	100	100	100	100
Total (ul)	1000	1000	1000	1000	1000	1000

Supporting Figure 2

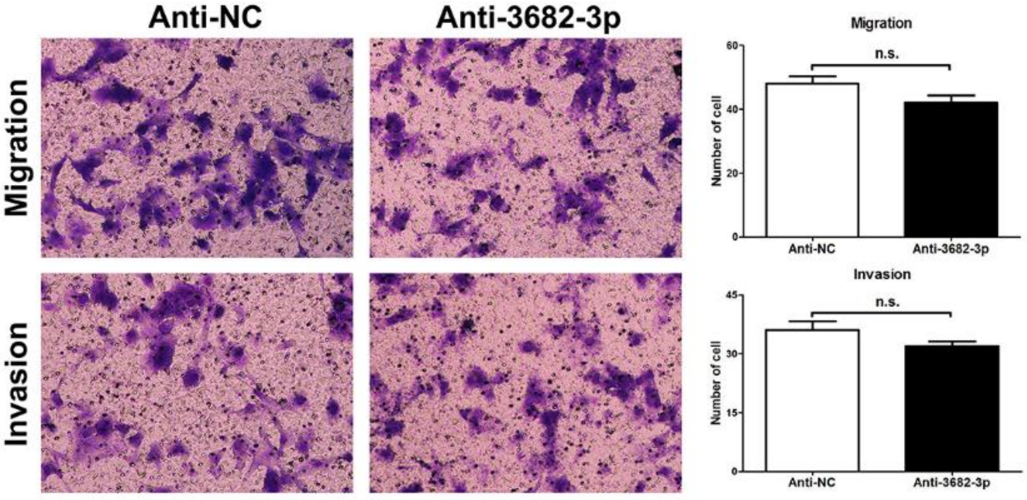

Supporting Figure 3

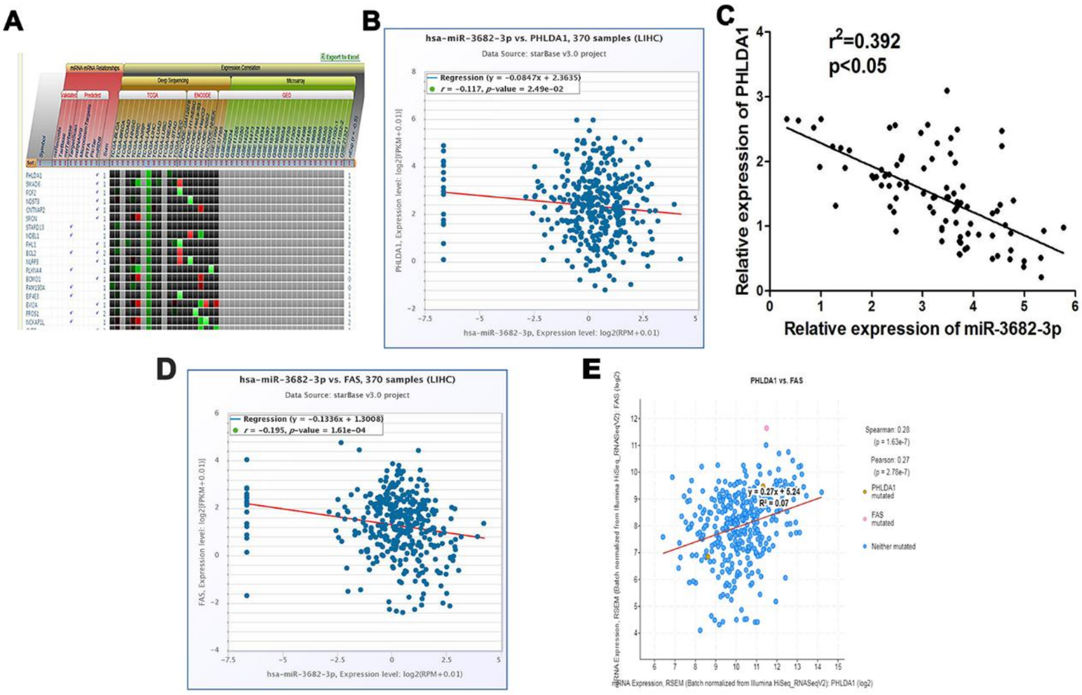

Supporting Figure 4

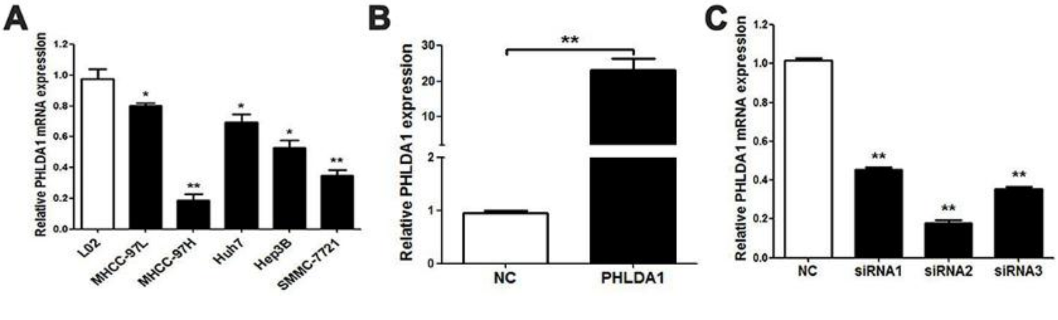

Supporting Figure 5

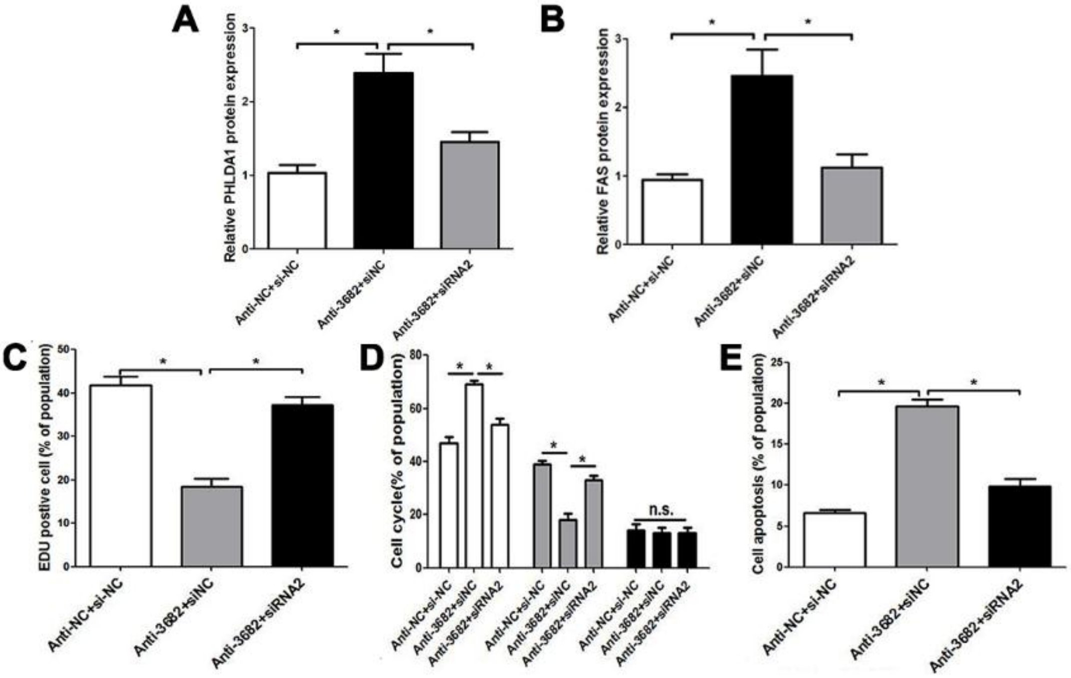

Supplement: Supplementary file 1 — Supplementary figures. [file jcav11p6188s1.pdf]
